# Small Alarmone Synthetase SasA Expression Leads to Concomitant Accumulation of pGpp, ppApp, and AppppA in *Bacillus subtilis*

**DOI:** 10.3389/fmicb.2020.02083

**Published:** 2020-09-02

**Authors:** Danny K. Fung, Jin Yang, David M. Stevenson, Daniel Amador-Noguez, Jue D. Wang

**Affiliations:** Department of Bacteriology, University of Wisconsin-Madison, Madison, WI, United States

**Keywords:** SasA, RelP, YwaC, pGpp, ppGpp, ppApp, AppppA, alarmone

## Abstract

(p)ppGpp is a highly conserved bacterial alarmone which regulates many aspects of cellular physiology and metabolism. In Gram-positive bacteria such as *B. subtilis*, cellular (p)ppGpp level is determined by the bifunctional (p)ppGpp synthetase/hydrolase RelA and two small alarmone synthetases (SASs) YjbM (SasB) and YwaC (SasA). However, it is less clear whether these enzymes are also involved in regulation of alarmones outside of (p)ppGpp. Here we developed an improved LC-MS-based method to detect a broad spectrum of metabolites and alarmones from bacterial cultures with high efficiency. By characterizing the metabolomic signatures of SasA expressing *B. subtilis*, we identified strong accumulation of the (p)ppGpp analog pGpp, as well as accumulation of ppApp and AppppA. The induced accumulation of these alarmones is abolished in the catalytically dead *sasA* mutant, suggesting that it is a consequence of SasA synthetase activity. In addition, we also identified depletion of specific purine nucleotides and their precursors including IMP precursors FGAR, SAICAR and AICAR (ZMP), as well as GTP and GDP. Furthermore, we also revealed depletion of multiple pyrimidine precursors such as orotate and orotidine 5′-phosphate. Taken together, our work shows that induction of a single (p)ppGpp synthetase can cause concomitant accumulation and potential regulatory interplay of multiple alarmones.

## Introduction

Survival of bacteria as single-cell organisms relies on their ability to adjust growth and cellular metabolism according to changes in the environment. A well-conserved mechanism to achieve such coordination is through the synthesis and degradation of the nucleotide alarmones ppGpp and pppGpp, collectively known as (p)ppGpp (Cashel and Gallant, 1969). (p)ppGpp regulates a repertoire of essential cellular processes including transcription, translation, ribosome synthesis, DNA replication, and nucleotide metabolism (Potrykus and Cashel, 2008; Liu et al., 2015a; Gourse et al., 2018), which altogether promotes stress adaptation and survival. In addition to (p)ppGpp, bacteria can produce a variety of other nucleotide alarmones such as pGpp, ppApp and AppppA (Bochner and Ames, 1982). However, the synthetases of these other alarmones and their roles in stress protection are less well understood. In addition, characterizing alarmones *in vivo* has been limited by the difficulty of profiling multiple alarmones in cell extracts.

In Firmicutes such as the pathogens *Enterococcus faecalis* and *Staphylococcus aureus*, or the soil bacterium *Bacillus subtilis*, (p)ppGpp can be produced by three different synthetases: the bifunctional synthetase/hydrolase Rel (traditionally called RelA in *B. subtilis*) (Wendrich and Marahiel, 1997), and two small alarmone synthetases YjbM (also known as SasB, RelQ, or SAS1) and YwaC (also known as SasA, RelP, or SAS2) (Nanamiya et al., 2008; Srivatsan et al., 2008; Geiger et al., 2014; Gaca et al., 2015). RelA is constitutively expressed and synthesizes (p)ppGpp by sensing starved ribosomes. SasB is also constitutively expressed but its (p)ppGpp synthesis activity is determined by allosteric activation by pppGpp (Steinchen et al., 2015) or single stranded RNA (Beljantseva et al., 2017).

In contrast to RelA and SasB, SasA expression is conditional and is regulated by the envelope stress sigma factors σ^M^ and σ^W^ (Cao et al., 2002). Unlike SasB, SasA from *S. aureus* does not require pppGpp binding for activation (Steinchen et al., 2018) but can be activated by low concentrations and inhibited by high concentrations of metal ions such as Zn^2+^ (Manav et al., 2018). Importantly, SasA expression can be induced by cell wall antibiotics to promote survival in response to drug treatment in *B. subtilis* and *S. aureus* (Geiger et al., 2014; Fung et al., 2020). However, the effect of SasA expression, without cell wall stress, on cellular alarmone and metabolome composition has not been characterized.

With the aim to investigate the characteristics of alarmone regulation by the cell wall stress induced (p)ppGpp synthetase SasA, we developed a LC-MS-based method to detect and measure an expanded set of metabolites and alarmones in *B. subtilis* cells with high efficiency. We found that SasA expression leads to strong accumulation of the (p)ppGpp analog pGpp, as well as accumulation of ppApp and AppppA to ∼10% of the level of pGpp. Furthermore, we also detected depletion of specific purine nucleotides and their precursors including GTP and GDP, and IMP precursors FGAR (Phosphoribosyl-N-formylglycineamide), SAICAR (Phosphoribosyl-aminoimidazolesuccinocarboxamide), and AICAR (5-Aminoimidazole-4-carboxamide ribonucleotide). Intriguingly, we revealed that SasA expression also leads to strong depletion of pyrimidine pathway precursors such as orotate and orotidine 5′-phosphate. Our work highlights that expression of SasA can cause concomitant accumulation of alarmones beyond (p)ppGpp, suggesting that regulation mediated by SasA involves multiple alarmones.

## Materials and Methods

### Bacterial Strains and Strain Construction

All bacterial strains, plasmids and oligonucleotides used in this study are listed in Table 1. LB and LB-agar were used for cloning and propagation of strains. For selection in *B. subtilis*, media was supplemented with the following antibiotics when necessary: spectinomycin (80 μg/mL), chloramphenicol (5 μg/mL), kanamycin (10 μg/mL), and a combination of lincomycin (12.5 μg/mL) and erythromycin (0.5 μg/mL) for MLS resistance. Carbenicillin (100 μg/mL) was used for selection in *E. coli*.

**TABLE 1:** Bacterial strains, plasmids, and oligonucleotides used in this study.

**Bacterial strains used in this study**			

**Strain**	**Organism**	**Genotype**	**Source**
JDW2901	*B. subtilis*	3610 Δ*zpdN* ΔSPβΔPBSX Δ*comI*	Daniel Kearns
BKK34780	*B. subtilis*	168 Δ*nahA*::*kan*^*R*^	BGSC
JDW3014	*B. subtilis*	JDW2901 Δ*ywaC*Δ*yjbM*	This work
JDW4009	*E. coli*	DH5α/pDR110-*ywaC*	This work
JDW4011	*E. coli*	DH5α/pDR110-*ywaC*^*D87G*^	This work
JDW4017	*B. subtilis*	JDW2901 Δ*ywaC*Δ*yjbM*Δ*relA::mls amyE::Pspank-ywaC*	This work
JDW4019	*B. subtilis*	JDW2901 Δ*ywaC*Δ*yjbM*Δ*relA::mls amyE::Pspank-ywaC^*D87G*^*	This work
JDW4064	*B. subtilis*	JDW2901 Δ*ywaC*Δ*yjbM*Δ*relA::mls amyE::Pspank-ywaC*Δ*nahA::kan^*R*^*	This work
JDW4066	*B. subtilis*	JDW2901 Δ*ywaC*Δ*yjbM*Δ*relA::mls amyE::Pspank-ywaC^*D87G*^*Δ*nahA::kan^*R*^*	This work

**Plasmids used in this study**			

**Plasmid**	**Genotype**		**Source**

pDR110	*amyE*::P*_*spank*_ amp spc*		David Rudner
pJW300	pJW239/Δ*yjbM* I-SceI site *amp cat*		Lab stock
pJW306	pJW299/Δ*ywaC* I-SceI site *amp cat*		Lab stock
pJW512	pDR154/*amyE*::P*_*xyl*_*-*ywaC*		Lab stock
pJW516	pDR154/*amyE*::P*_*xyl*_*-*ywaC*^*D87G*^		Lab stock
pJW731	pDR110/*amyE*::P*_*spank*_*-*ywaC*		This work
pJW733	pDR110/*amyE*::P*_*spank*_*-*ywaC*^*D87G*^		This work
pSS4332	*oriU* P*_*amy*_*-I-sceI *kan*		Scott Stibitz

**Oligonucleotides used in this study**			

**Oligo**	**Sequence (5′ 3′)**		

oJW358	ATGTATGGCCGGAACTGAAG		
oJW359	CGGTCGCTGTATCTGTGAAA		
oJW902	AAAGAGGCGCTTTTGACGTG		
oJW903	TTGTTGACCCGGGACATGGA		
oJW904	CGTCCTCATACGTTAACCGC		
oJW905	GGGCTATCAAAAGGACTTTACCG		
oJW1519	TCATCCATCATACATCCTCCTTCTGTCGACTTAATTAAACCACTTTG		
oJW3382	GCTCAAAGTATTCTTCAAGCGAGAG		
oJW3383	CATTCCACTTCATGACGTAAGAGG		
oJW3495	ACATGCATGCAAAGGAGGTGTACATATGGATTTATCTGTAACACATATGGACG		
oJW3496	ACATGTCGACTTAATCCACTTCTTTCTTAATCCCCAGC		

Construction of pJW731 and pJW733 was done by PCR amplification of *ywaC* and *ywaC*^*D87G*^ fragments with primers oJW3495/3496 using pJW512 and pJW516 as templates, followed by *Sal*I/*Sph*I digestion and ligation into pDR110. The resulting plasmid is transformed into *E. coli* DH5α for propagation and verified by sequencing with oJW1519.

Construction of JDW3014 was done by sequential transformations of integration plasmids containing an I-sceI endonuclease cut site and regions of homology upstream and downstream of synthetase genes (pJW300 for Δ*yjbM* and pJW306 for Δ*ywaC*) followed by transformation of pSS4332 for marker-less recombination (Janes and Stibitz, 2006). Successful removal of the synthetase genes was verified by PCR and sequencing (oJW358/359 for *yjbM* and oJW904/905 for *ywaC*).

Construction of JDW4017 and JDW4019 was done by integration of JDW3014 at *amyE* with pJW731 and pJW733, followed by selection for spectinomycin resistance. The resulting strain was then transformed with Δ*relA*::*mls* PCR product synthesized from genomic DNA using oligos oJW902/oJW903, followed by selection for MLS resistance (Kriel et al., 2012). Disruption of *relA* was verified by PCR and sequencing (oJW418/419).

Construction of JDW4064 and JDW4066 was done by integration of JDW3014 at *amyE* with pJW731 and pJW733, followed by selection for spectinomycin resistance. The resulting strain was transformed with Δ*nahA*::*kan*^*R*^ PCR product synthesized from genomic DNA of BKK34780 (BGSC) using oligos oJW3382/oJW3383, followed by selection for kanamycin resistance. The resulting strain was further transformed with Δ*relA*::*erm*^*R*^ PCR product synthesized from genomic DNA using oligos oJW902/oJW903, followed by selection for MLS resistance (Kriel et al., 2012). Disruption of *nahA* and *relA* was verified by PCR and sequencing (oJW3382/oJW3383 for *nahA* and oJW418/419 for *relA*).

### Growth Conditions

*Bacillus subtilis* strains were grown in S7 defined medium (Harwood and Cutting, 1990); MOPS was used at 50 mM rather than 100 mM, supplemented with 0.1% glutamate, 1% glucose, and 20 amino acids (50 μg/mL alanine, 50 μg/mL arginine, 50 μg/mL asparagine, 50 μg/mL glutamine, 50 μg/mL histidine, 50 μg/mL lysine, 50 μg/mL proline, 50 μg/mL serine, 50 μg/mL threonine, 50 μg/mL glycine, 50 μg/mL isoleucine, 50 μg/mL leucine, 50 μg/mL methionine, 50 μg/mL valine, 50 μg/mL phenylalanine, 500 μg/mL aspartic acid, 500 μg/mL glutamic acid, 20 μg/mL tryptophan, 20 μg/mL tyrosine, and 40 μg/mL cysteine). Cells were harvested from young, overnight LB-agar plates (< 12 h), back-diluted into fresh S7 defined media at OD_600_ = 0.005, and grown at 37°C with vigorous shaking to logarithmic phase (OD_600_ ≈ 0.1–0.3). Induction of SasA expression was done by addition of 1 mM IPTG at final concentration. Cell viability assay was done by serial dilution and plating on LB plates, followed by colony counting after overnight incubation at 37°C.

### Sample Collection and LC-MS Quantification of Nucleotides

LC-MS quantification of nucleotides was performed as described (Liu et al., 2015b) with modifications (Yang et al., 2020). Cells were grown in S7 defined medium to OD_600_ ∼0.3 followed by addition of 1 mM IPTG. For sample collection, 10 mL cultures were sampled by filtering through PTFE membrane (Sartorius) before and after 30-min IPTG induction. Filtered membranes with harvested cells were immediately submerged in 3 mL extraction solvent mix [on ice 50:50 (v/v) chloroform/water] to quench metabolism. This process also enables efficient cell lysis and extraction of soluble metabolites. Mixture of cell extracts were centrifuged at 5000 × g for 10 min to remove organic phase, then centrifuged at 20,000 × g for 10 min to remove cell debris. Samples were frozen at −80°C if not analyzed immediately. Samples were analyzed using HPLC-MS system consisting of a Vanquish UHPLC system linked to electrospray ionization (ESI, negative mode) to a Q Exactive Orbitrap mass spectrometer (Thermo Scientific) operated in full-scan mode to detect targeted metabolites based on their accurate masses. LC was performed on an Acquity UPLC BEH C18 column (1.7 μm, 2.1 × 100 mm; Waters). Total run time was 30 min with a flow rate of 0.2 mL/min, using Solvent A [97:3 (v/v) water/methanol, 10 mM tributylamine and 10 mM acetic acid] and acetonitrile as Solvent B. The gradient was as follows: 0 min, 5% B; 2.5 min, 5% B; 19 min, 100% B; 23.5 min 100% B; 24 min, 5% B; 30 min, 5% B.

### Data Analysis

Quantification of metabolites from raw LC-MS data were performed by using the MAVEN software (Clasquin et al., 2012). Metabolite levels of different samples were normalized to their respective OD_600_ to the same sample volumes (10 mL). Prism 7 (GraphPad) was used for statistical analysis and generation of figures.

### Calculation of Metabolite Concentrations

We use the estimation that cell volume is 0.475 μL in 1 mL culture at an OD_600_ of 1.0. We adopt a cell density of 2.0 × 10^8^ CFU/mL/OD_600_ and the shape of cytoplasm as a cylinder of 4 μm in height and 0.435 μm in radius in the calculation. This estimation corresponds to an average cell volume of 2.38 fL.

The detection efficiency of pGpp in LC-MS is around 2.4e8 ion counts/μM in 25 μL sample. Normalized ion count can be converted into intracellular concentration of pGpp by $C_{P\,3\,G}=\frac{N\,I_{P\,3\,G}}{E_{L\,C-M\,S}\times V_{C\,u\,l\,t\,u\,r\,e,N}\times O\,D_{600\,n\,m,N}\times F_{c\,e\,l\,l}}$, where *NI*_*P3G*_ is the normalized ion count of pGpp, *E*_*LC–MS*_ is the detection efficiency of pGpp in LC-MS (2.4e8 ion counts/μM), *V*_*Culture,  N*_ is the normalized culture volume (5.0 mL), *OD*_600*nm*,*N*_is the normalized optical density (1.0) and *F*_*cell*_ is the fraction of cell volume in the culture (0.000475 mL/1 mL culture/OD).

The detection efficiency of other nucleotides in LC-MS is around 2.0e8 ion counts/μM in 25 μL sample. Normalized ion count can be converted into intracellular concentration of nucleotide by $C_{n\,t}=\frac{N\,I_{n\,t}}{E_{L\,C-M\,S}\times V_{C\,u\,l\,t\,u\,r\,e,N}\times O\,D_{600\,n\,m,N}\times F_{c\,e\,l\,l}}$, where *NI*_*nt*_ is the normalized ion count of nucleotide, *E*_*LC–MS*_ is the detection efficiency of nucleotide in LC-MS (2.0e8 ion counts/μM), *V*_*Culture,  N*_ is the normalized culture volume (5.0 mL), *OD*_600*nm*,*N*_is the normalized optical density (1.0) and *F*_*cell*_ is the fraction of cell volume in the culture (0.000475 mL/1 mL culture/OD).

## Results

### Development of an Improved LC-MS Method for Alarmone Detection in *B. subtilis*

**FIGURE 1:**
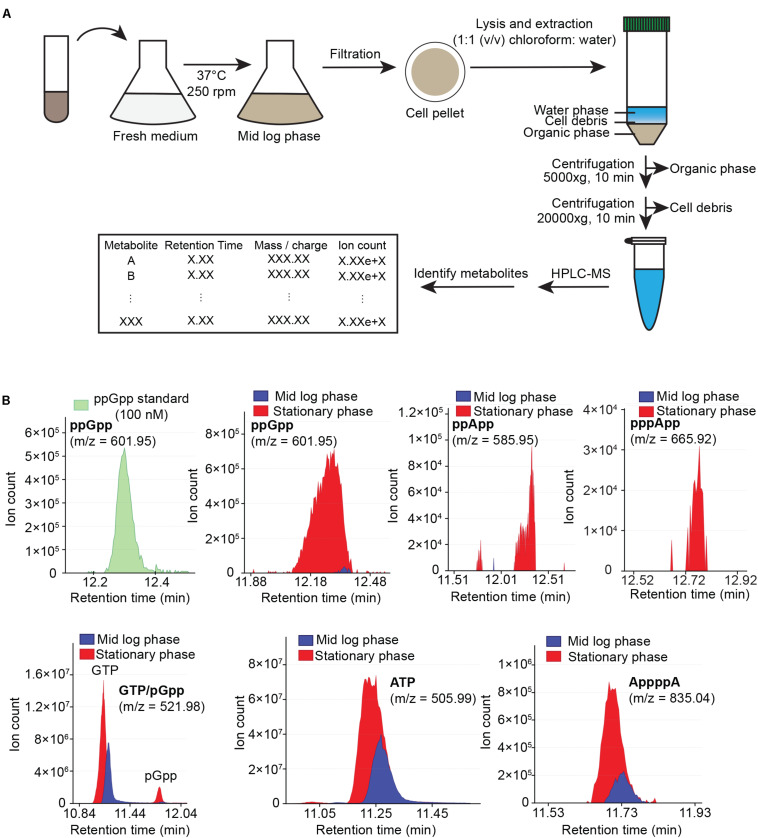
Cellular metabolites extraction and analysis using HPLC-coupled mass spectrometry (HPLC-MS). **(A)** Flow chart of sample preparation, metabolite extraction and analysis. **(B)** HPLC-MS profile of ppGpp standard (green), and sample HPLC-MS profiles of ppGpp, ppApp, pppApp, GTP, pGpp, ATP, and AppppA detected in mid log phase (blue) and stationary phase (red) *B. subtilis* grown in minimal media. Data shown are raw ion counts.

Our previous metabolite extraction and LC-MS analysis method allowed us to efficiently detect and quantitate high abundance metabolites such as GTP in *B. subtilis* (Liu et al., 2015b), however, we were unable to detect alarmones such as (p)ppGpp even in starvation-induced *B. subtilis*. Upon revisiting our LC-MS analysis protocol, we found that pure (p)ppGpp can be sensitively detected by LC-MS at concentrations > 10 nM with 100% acetonitrile as the buffer B, but not from *B. subtilis* cell extracts using the same method (data not shown). This suggests that the lack of (p)ppGpp signals from bacterial samples was due to inefficient metabolite extraction procedures. To this end, we optimized the method to increase the breadth of detectable metabolites from cell extracts. We improved our metabolite extraction procedures (Figure 1A) by replacing hydrophilic nylon filtration filters with hydrophobic PTFE filters, as well as using 1:1 (v/v) chloroform: water for lysis and extraction instead of 40:20:20 (v/v/v) acetonitrile/methanol/water. These modifications allowed improved recovery of alarmones by preventing adsorption of (p)ppGpp by the nylon membrane and increasing (p)ppGpp solubility in the extraction solvent. Furthermore, we used acetonitrile instead of methanol for solvent B in liquid chromatography which improved the resolution of low abundance metabolites such as alarmones. We found that our improved LC-MS protocol allowed us to sensitively detect alarmones such as pGpp, ppGpp, pppGpp, ppApp, pppApp, and AppppA from cell extracts, while retaining detection capability for other nucleotides such as ATP and GTP (Figure 1B). While we detect significant amount of ppGpp in mid log phase *B. subtilis* grown in minimal media, we found that (p)ppGpp in mid log phase *B. subtilis* grown in rich media is below detection limit, likely due to the extremely low concentration of (p)ppGpp in this condition.

### Expression of SasA Leads to Accumulation of Multiple Alarmones

**FIGURE 2:**
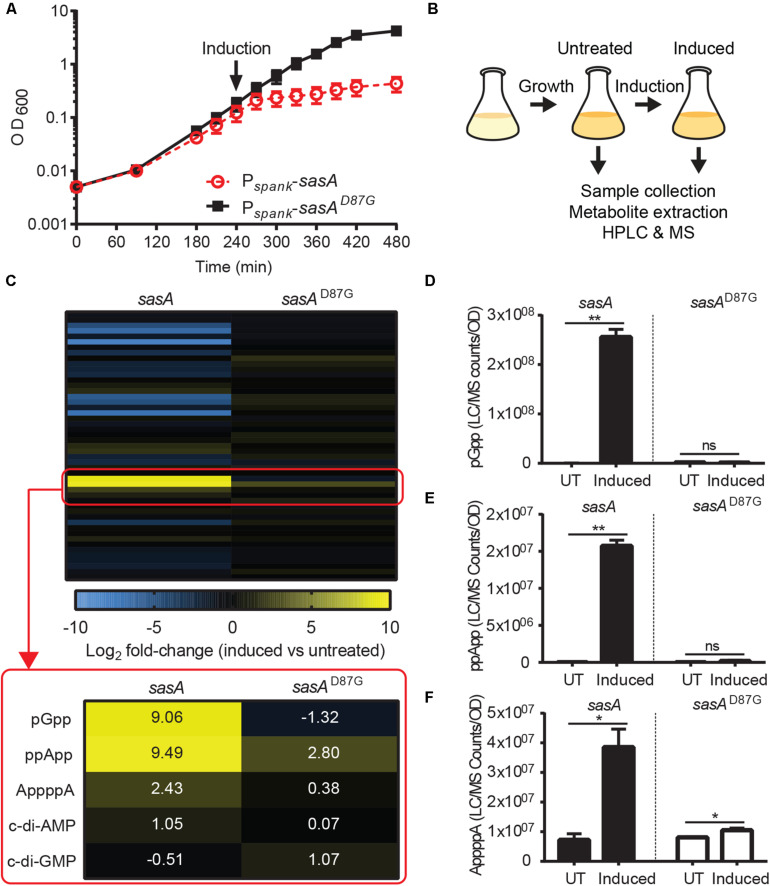
SasA expression leads to accumulation of pGpp, ppApp, and AppppA. **(A)** Growth of *sasA* (solid square) and *sasA*^*D87G*^ (open circle) expression strains measured by OD_600_. *sasA*^*D87G*^ encodes a synthetase-dead variant of SasA. Arrow indicates induction with 1 mM IPTG. **(B)** Schematic of metabolome profiling experiment. Cultures were grown to OD_600_∼0.3 followed by 30 min IPTG induction. Cells before and after 30 min IPTG induction were immediately harvested for metabolite extraction and HPLC-MS analysis as described in Figure 1. **(C)** Heat map of metabolite changes in cells after *sasA* or *sasA*^*D87G*^ expression. Red box highlights the increases in the level of alarmones and other detected signaling molecules. Numbers indicate mean fold-change in binary logarithm relative to untreated cells. **(D–F)** Levels of **(D)** pGpp, **(E)** ppApp and **(F)** AppppA in cells before and after *sasA* expression. UT: untreated, Induced: after induction. Data shown are LC-MS ion counts normalized to OD_600_. Error bars indicate SD. *n* = 2. **p < 0.01, *p < 0.05, ns, not significant (Student’s *t*-test).

Next, we applied our improved LC-MS detection method to investigate the metabolomic signatures upon SasA expression. It is known that SasA is transcriptionally induced by cell envelope perturbations due to alkaline stress (Nanamiya et al., 2008) or antibiotics (Cao et al., 2002). To understand the primary effects of SasA expression and to avoid alarmone synthesis by other (p)ppGpp synthetases, we constructed a strain that ectopically expresses an IPTG-inducible SasA or SasA^D87G^ (synthetase-dead SasA) in the absence of the other two (p)ppGpp synthetases RelA and SasB. Because *B. subtilis* without (p)ppGpp production is auxotrophic for multiple amino acids (Kriel et al., 2014), we grew the strains in rich media to minimize growth defect and suppressors. Both strains grew at similar rates prior to induction (Figure 2A), implying little basal activity from potential leaky expression. Upon induction, the SasA over-expression strain stopped growth in ∼30 min while the *sasA*^D87G^ mutant was unaffected (Figure 2A), confirming accumulation of alarmones in the SasA over-expression strain. The induction also had no effect on cell viability even after prolonged induction (Supplementary Figure S1), excluding confounding effects due to cell death.

Using metabolomics analysis, we found that SasA expression alone can cause profound changes in cellular levels of alarmones, nucleotides and their precursors (Figure 2B and Supplementary Figure S2). We found that expression of SasA resulted in strong increase in the levels of alarmones pGpp, ppApp and AppppA (Figure 2C). These increases are abolished in SasA^*D87G*^ cells (Figure 2C), indicating that they are dependent on the (p)ppGpp synthetase activity of SasA. The most strongly induced alarmone is pGpp, a (p)ppGpp analog, reaching up to ∼2.5 × 10^8^ normalized LC-MS counts (Figure 2D) which roughly converts to ∼0.3 mM in the cell. The high level of pGpp is also due to failure of its hydrolysis by RelA due to RelA deletion.

**FIGURE 3:**
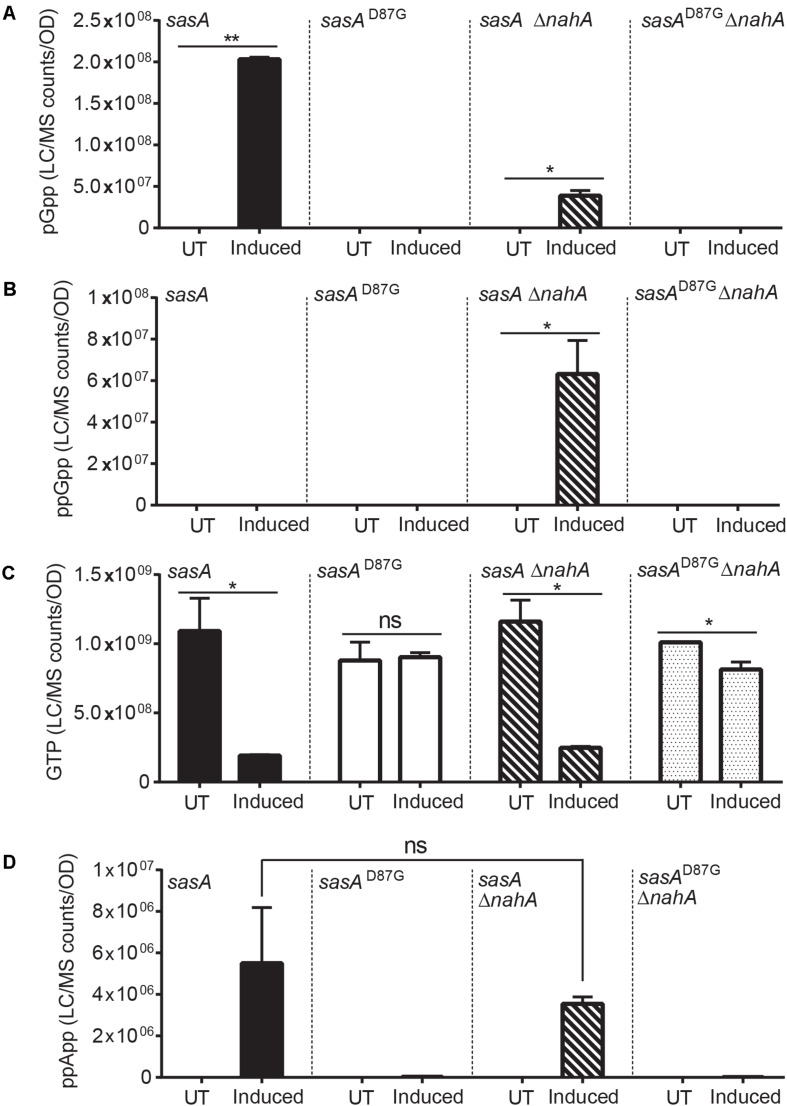
Production of pGpp after induction of SasA is largely mediated by NahA. Bar plots of **(A)** pGpp, **(B)** ppGpp, **(C)** GTP, and **(D)** ppApp levels before and after *sasA* expression in cells with or without *nahA*. UT: untreated, Induced: after induction. Data shown are LC-MS ion counts normalized to OD_600_. Error bars indicate *SD*. *n* = 2. **p < 0.01, *p < 0.05, ns, not significant (Student’s *t*-test).

Intriguingly, the level of pppGpp and ppGpp were both below the detectable range. However, this is not unexpected, because ppGpp synthesized by SasA can be rapidly converted to pGpp by the newly discovered (p)ppGpp hydrolyzing enzyme NahA (Yang et al., 2020). To test this hypothesis, we measured ppGpp, pGpp and GTP levels in SasA and SasA^D87G^-expressing cells in the *nahA* mutant (Figure 3). We found that deletion of *nahA* led to a significant decrease (∼80%) in pGpp (Figure 3A) along with a strong increase in ppGpp (Figure 3B). This demonstrates that ppGpp is a major product of SasA but is efficiently converted to pGpp by NahA. On the other hand, changes in other metabolites such as GTP were unaffected (Figure 3C). In addition, we found that there is a low level of pGpp detected even in the Δ*nahA* mutant, suggesting that some pGpp can be directly produced by SasA, or there is another hydrolase which can convert ppGpp to pGpp in *B. subtilis*.

In addition to pGpp, we detected strong accumulation of another nucleotide alarmone ppApp to a level ∼10% of that of pGpp (Figure 2E). Unlike pGpp, ppApp level was unaffected by NahA (Figure 3D). Intriguingly, *in vitro* evidence suggest that SasA from *S. aureus* can directly produce ppApp and pppApp (Wieland Steinchen & Gert Bange, personal communication). Therefore, it is likely that SasA can synthesize ppApp as an alternative product in *B. subtilis*.

Furthermore, we detected a ∼4-fold increase in AppppA upon SasA induction (Figure 2F) to a level similar to that of ppApp. AppppA is not known to be a product of SasA, thus its accumulation can be due to indirect effects resulting from increased availability of its precursors (see below and “Discussion”). Taken together, these results suggest that expression of SasA can lead to accumulation of multiple alarmones in addition to its expected product.

### Alarmone Synthesis by SasA Results in Reduction of Guanine Nucleotides and Accumulation of Adenine Nucleotides in *B. subtilis*

**FIGURE 4:**
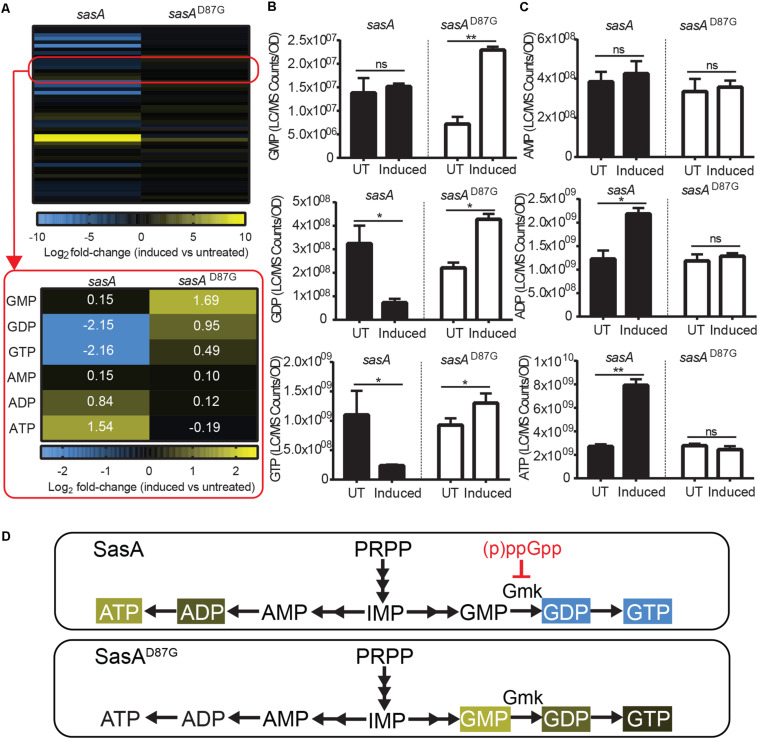
Expression of SasA leads to depletion of guanine nucleotides and accumulation of adenine nucleotides. **(A)** Heat map of metabolite changes in cells after *sasA* or *sasA*^*D87G*^ expression. Red box highlights the changes in the level of guanine and adenine nucleotides. GMP, Guanosine monophosphate; GDP, Guanosine diphosphate; GTP, Guanosine triphosphate; AMP, Adenosine monophosphate; ADP, Adenosine diphosphate; ATP, Adenosine triphosphate. Numbers indicate mean fold-change in binary logarithm relative to untreated cells. **(B)** Levels of GMP, GDP, and GTP in cells before and after *sasA* or *sasA*^*D87G*^ expression. **(C)** Levels of AMP, ADP, and ATP in cells before and after *sasA* or *sasA*^*D87G*^ expression. UT: untreated, Induced: after induction. Data shown are LC-MS ion counts normalized to OD_600_. Error bars indicate *SD*. *n* = 2. **p < 0.01, *p < 0.05, ns, not significant (Student’s *t*-test). **(D)** Summary of nucleotide changes in cells expressing *sasA* or *sasA*^*D87G*^. Color-shaded are metabolites with log_2_ fold-change ≥ 0.5 (yellow) or = −0.5 (blue) as shown in **(A)**. Red blunt arrow indicates direct inhibition by (p)ppGpp. Gmk: Guanylate kinase.

In addition to accumulation of alarmones, we identified changes in the levels of purine nucleotides upon SasA expression (Figure 4A). We detected significant decreases of GDP (∼4-fold) and GTP (∼4-fold), while the level of GMP remain unchanged (Figure 4B). These changes were attenuated or reversed in the SasA^D87G^-expressing cells (Figure 4B). For adenine nucleotides, while AMP remained largely unchanged, we detected significant increases in ADP (∼1.8-fold) and ATP (∼3-fold) upon SasA expression. In contrast, no significant changes in AMP, ADP or ATP were observed in the *sasA*^D87G^ mutant (Figure 4C). The changes in GTP and ATP pools correspond to an estimated decrease in GTP from ∼1.7 mM to ∼0.4 mM and an estimated increase in ATP from ∼4.3 to ∼12.5 mM. In summary, we observed an overall reduction of guanine nucleotides and accumulation of adenine nucleotides during SasA-mediated alarmone accumulation (Figure 4D).

**FIGURE 5:**
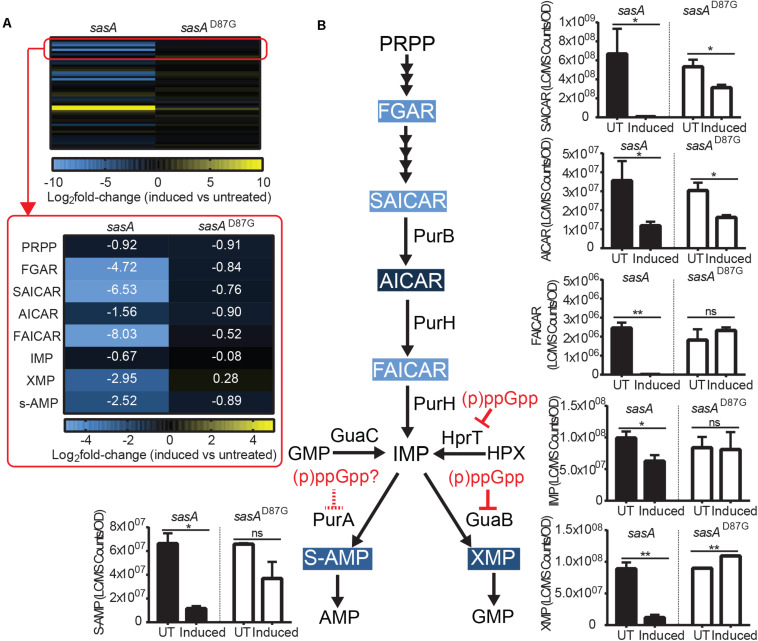
Purine precursors are depleted during SasA expression. **(A)** Heat map of metabolite changes in cells after *sasA* or *sasA*^*D87G*^ expression. Red box highlights the changes in the level of PRPP-IMP pathway metabolites in addition to adenylosuccinate (S-AMP) and xanthosine monophosphate (XMP). PRPP, Phosphoribosyl pyrophosphate; FGAR, Phosphoribosyl-N-formylglycineamide; SAICAR, Phosphoribosylaminoimidazolesuccinocarboxamide; AICAR, 5-Aminoimidazole-4-carboxamide ribonucleotide; FAICAR, 5-Formamidoimidazole-4-carboxamide ribotide; IMP, Inosine monophosphate. Numbers indicate mean fold-change in binary logarithm relative to untreated cells. **(B)** Summary of metabolite changes in the *de novo* purine biosynthesis pathway in SasA-expressing cells. Bar plots shown are levels of SAICAR, AICAR, FAICAR, IMP, S-AMP, and XMP before and after *sasA* or *sasA*^*D87G*^ expression. PurB, Adenylsuccinate lyase; PurH, Phosphoribosylaminoimidazole carboxamide formyltransferaseand inosine-monophosphate cyclohydrolase; GuaC, GMP reductase; HprT, Hypoxanthine phosphoribosyltransferase; PurA, Adenylosuccinate synthetase; GuaB, IMP dehydrogenase. UT: untreated, Induced: after induction. Data shown are LC-MS ion counts normalized to OD_600_. Error bars indicate *SD*. *n* = 2. **p < 0.01, *p < 0.05, ns: not significant (Student’s *t-*test). Color-shaded are metabolites with log_2_ fold-change > 2 as shown in **(A)**. Solid and dashed red arrows indicate known and speculated inhibition by (p)ppGpp respectively.

AMP and GMP are synthesized from S-AMP (adenylosuccinate) and XMP respectively using IMP as a common precursor (Figure 5B). We found that the level of IMP and its salvage pathway precursor HPX were only mildly reduced by ∼30–40% (Figure 5B and Supplementary Figure S3) after induced wild type SasA expression. However, we detected strong reductions of both S-AMP (∼5-fold) and XMP (∼7-fold) (Figure 5B), possibly due to direct enzymatic inhibition of PurA (adenylosuccinate synthetase) and GuaB (IMP dehydrogenase) by the alarmones.

#### *De novo* IMP Synthesis Pathway Intermediates Are Depleted During SasA Expression

The most drastic changes in metabolites we observed upon SasA expression are in the *de novo* IMP biosynthesis pathway (Figure 5A). Although we found no detectable difference in PRPP levels upon SasA expression (Figure 5A), we found profound changes in IMP precursors phosphoribosyl-N-formylglycineamide (FGAR), phosphoribosyl-aminoimidazolesuccinocarboxamide (SAICAR), 5-Aminoimidazole-4-carboxamide ribonucleotide (AICAR) and 5-Formamidoimidazole-4-carboxamide ribotide (FAICAR) which are produced at different steps in the PRPP-IMP pathway. In the SasA^D87G^ expression strain, we detected only mild changes (< 2-fold) in these metabolites before and after induction (Figure 5A). In contrast, expression of SasA resulted in strong depletion of FAICAR (> 100-fold), SAICAR (> 100-fold) and FGAR (∼30-fold), as well as ∼3-fold reduction in AICAR (Figures 5A,B). This suggests a strong inhibitory effect of alarmones on the synthesis of multiple PRPP-IMP pathway intermediates. However, enzymes in the PRPP-IMP pathway have not been found to be direct targets of (p)ppGpp in *B. subtilis*, suggesting the inhibition can be mediated by other alarmones or through modulation of their expression. Taken together, our data showed that SasA expression resulted in overall depletion of IMP synthesis precursors (Figure 5B).

### *De novo* Pyrimidine Nucleotide Synthesis Pathway Intermediates Are Drastically Changed During SasA Expression

**FIGURE 6:**
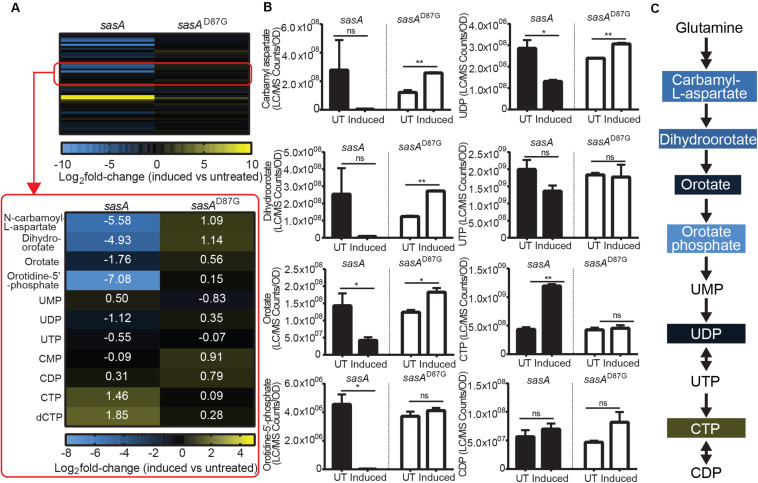
SasA expression leads to depletion of pyrimidine precursors and increase in CTP. **(A)** Heat map of metabolite changes in cells after *sasA* or *sasA*^*D87G*^ expression. Red box highlights the changes in the level of pyrimidine pathway metabolites. UMP, Uridine monophosphate; UDP, Uridine diphosphate; UTP, Uridine triphosphate; CMP, Cytidine monophosphate; CDP, Cytidine diphosphate; CTP, Cytidine triphosphate; dCTP, Deoxycytidine triphosphate. Numbers indicate mean fold-change in binary logarithm relative to untreated cells. **(B)** Bar plots of pyrimidine pathway metabolites before and after *sasA* or *sasA*^*D87G*^ expression. UT: untreated, Induced: after induction. Data shown are LC-MS ion counts normalized to OD_600_. Error bars indicate *SD*. *n* = 2. **p < 0.01, *p < 0.05, ns, not significant (Student’s *t*-test). **(C)** Summary of metabolite changes in the pyrimidine pathway in *sasA*-expressing cells. Color-shaded are metabolites with log_2_ fold-change > 1 as shown in **(A)**.

In addition to changes in the levels of purine nucleotides and its precursors, we also observed perturbations of the *de novo* pyrimidine synthesis pathway during SasA expression (Figure 6A). We found drastic changes in the levels of pyrimidine nucleotide precursors (Figure 6A). Apart from the undetectable carbamoyl-phosphate and glutamine which is supplied in growth media, all other four intermediates including N-carbamoyl-L-aspartate, dihydroorotate, orotate, and orotidine-5′-phosphate were depleted by ∼3.4-fold (orotate) to ∼135-fold (orotidine-5′-phosphate) after the induction of SasA expression (Figure 6B). In addition, we identified higher CTP (∼2.7 fold), dCTP (∼3.6-fold), and UMP (∼1.8 fold) levels after SasA induction (Figure 6A). UDP was slightly decreased by ∼2.2-fold (Figure 6B). These changes were abolished or reversed in the *sasA*^D87G^ mutant (Figures 6A,B). These data showed that SasA expression resulted in remodeled pyrimidine nucleotide abundance and depletion of *de novo* pyrimidine synthesis precursors (Figure 6C).

## Discussion

**FIGURE 7:**
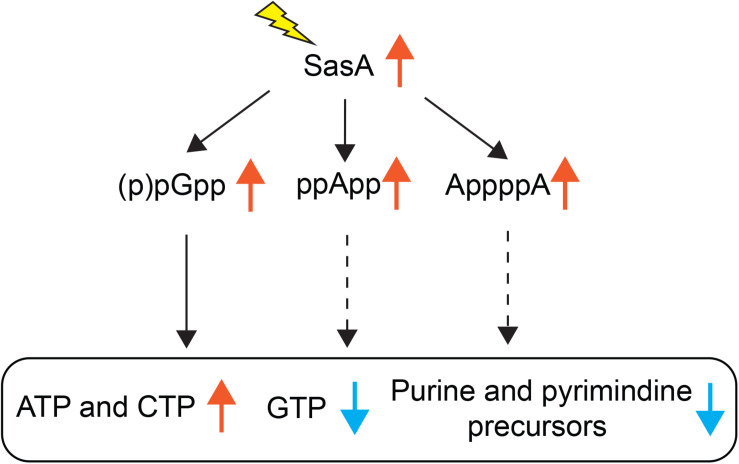
Effects of SasA expression on alarmones and nucleotide synthesis. Expression of SasA in the absence of other (p)ppGpp synthetases leads to concomitant accumulations of (p)ppGpp analogs pGpp, ppApp and AppppA, as well as depletions in GTP and purine precursors. To the contrary, ATP level is increased. Furthermore, pyrimidine precursors are also depleted along with increases in CTP. Our recent findings on pGpp binding targets (Yang et al., 2020) suggest that pGpp targets are largely similar to that of (p)ppGpp. Thus, the depletion of GTP and inhibition of purine precursor biosynthesis is likely a downstream effect of pGpp (solid arrows). The roles of ppApp or AppppA on nucleotide biosynthesis regulation (dashed arrows) remains to be determined.

Understanding the breadth of alarmone regulation by (p)ppGpp synthetases is important to understand their roles in cellular physiology. However, precise detection and quantitation of alarmones in bacteria has been challenging until recent advances in MS-based detection methods (Varik et al., 2017; Zbornikova et al., 2019). Here we documented an improved LC-MS protocol that allows efficient detection and quantitation of multiple alarmones and metabolites in *B. subtilis* cells. Using this method, we studied the metabolic signatures of stringent response mediated by the small alarmone synthetase SasA which is transcriptionally induced in response to cell wall stresses. Apart from increased level of the (p)ppGpp derivative pGpp, we detected unexpected accumulations of ppApp, AppppA and a mild increase of other signaling molecules such as c-di-AMP, as well as changes in *de novo* purine and pyrimidine biosynthesis metabolites (Figure 7). Our findings suggest that expression of (p)ppGpp synthetase can affect the levels of alarmones outside of (p)ppGpp, implying complex multi-alarmone regulations during the stringent response.

### Accumulation of Multiple Alarmones From SasA Expression

The increase in multiple alarmones and second messengers upon SasA expression is likely due to both direct and indirect mechanisms. First, SasA may produce ppApp and pGpp in addition to (p)ppGpp. Although it was showed that SasA from *S. aureus* can efficiently synthesize ppGpp *in vitro* (Steinchen et al., 2018), it is possible that the enzyme can produce other (p)ppGpp analogs such as pGpp and ppApp depending on substrate availability. For example, another small alarmone synthetase SasB in *Enterococcus faecalis* can produce pGpp *in vitro* (Gaca et al., 2015). On the other hand, Rel from *Methylobacterium extorquens* can synthesize pppApp both *in vitro* and when expressed in *E. coli* (Sobala et al., 2019), and the secreted toxin Tas1 from *Pseudomonas aeruginosa* can produce pppApp, ppApp, and pApp in target *E. coli* cells to mediate contact dependent inhibition (Ahmad et al., 2019). These findings suggest that stress responses mediated by (p)ppGpp synthetases may not be strictly limited to the alarmones pppGpp and ppGpp.

Second, pGpp can be produced efficiently from (p)ppGpp through the NuDiX hydrolase NahA (Yang et al., 2020). We found that under SasA expression the majority of accumulated pGpp was due to NahA-mediated conversion of ppGpp produced by SasA (Figures 3A,B). On the other hand, we could also detect a low level of pGpp in the *nahA* mutant, suggesting that SasA may directly produce pGpp (Figures 3A,B). However, we cannot rule out the presence of another hydrolase in *B. subtilis* which can convert ppGpp to pGpp.

AppppA is known to be produced from ATP through distinct mechanisms catalyzed by aminoacyl-tRNA synthetases (Supplementary Figure S4; Bochner et al., 1984). Due to the disparity between these enzymes and (p)ppGpp synthetases, the possibility that SasA can directly synthesize AppppA is low. Instead, one plausible explanation to its accumulation is through indirect increases of ATP (Figure 4C) which is the initiating substrate for its synthesis. Our characterization thus supports the interconnected nature of purine nucleotides, not just for guanine, but also for adenine nucleotides.

### Depletion of GTP and Purine Precursors by Alarmone Accumulation

Apart from alarmone accumulation, we found that SasA expression also resulted in depletion of GTP and accumulation of ATP. This characteristic metabolic change resembles the metabolic changes during amino acid starvation (Kriel et al., 2012) which is primarily mediated by the (p)ppGpp synthetase-hydrolase RelA. Although we detected pGpp and ppApp instead of (p)ppGpp as the predominant alarmones synthesized by SasA, the drop in GTP is at least contributed by direct inhibition of the GMP kinase Gmk by pGpp, similarly to that of (p)ppGpp (Liu et al., 2015b). This is supported by recent finding that pGpp, ppGpp, and pppGpp share similar binding properties to *de novo* purine biosynthesis enzymes (Yang et al., 2020). Whether the other (p)ppGpp analog ppApp can directly regulate GTP synthesis is under investigation.

In addition, we also identified depletion of both S-AMP (adenylosuccinate) and XMP which are products of IMP. Synthesis of XMP from IMP is catalyzed by GuaB which has been reported to interact with ppGpp in *E. coli* (Zhang et al., 2018) but is only weakly inhibited by (p)ppGpp in *B. subtilis* (Kriel et al., 2012). The strong inhibition of XMP synthesis observed here suggests that the inhibition can potentially be mediated by alternative mechanisms, such as through other induced alarmones or by repression of *guaB* expression. On the other hand, S-AMP synthesis from IMP is catalyzed by PurA (Saxild and Nygaard, 1991) which is also a ppGpp-binding target in *E. coli* (Pao and Dyess, 1981). Consistently, we have recently identified PurA as a target of (p)ppGpp and pGpp in *B. anthracis* (Yang et al., 2020), suggesting that the inhibition of S-AMP synthesis is at least partially attributed to pGpp.

Interestingly, we revealed that intermediates (e.g., FGAR, SAICAR, and FAICAR) in the upstream PRPP-IMP pathway are also depleted in response to SasA expression. Since PRPP level was similar, the inhibition is likely specific to the catalytic steps between PRPP and IMP which are catalyzed by the gene products of the *pur* operon. While none of the enzymes from this operon have been found to be a (p)ppGpp target in *B. subtilis*, we have previously found that transcription of the *pur* operon is strongly downregulated during starvation in a (p)ppGpp-dependent manner (Kriel et al., 2014). This is supported by our recent finding that the *pur* operon repressor PurR can bind to pGpp and (p)ppGpp (Yang et al., 2020). Thus, it is possible that regulation of purine synthesis by (p)ppGpp in *B. subtilis* involves two distinct components: transcription control for upstream intermediates and direct interaction for downstream precursors. This is in contrast to regulation in *E. coli* where a number of PRPP-IMP pathway enzymes such as PurF, PurB, and PurC have been recently identified as targets of (p)ppGpp (Wang et al., 2019), which highlights the disparity of purine synthesis control between evolutionarily distant species.

### Physiological Implications of SasA-Mediated Growth Control

Unlike (p)ppGpp synthesis triggered by nutrient starvation through the (p)ppGpp synthetase RelA, (p)ppGpp synthesis by SasA is induced in response to cell wall stresses (Nanamiya et al., 2008; Geiger et al., 2014). The physiological benefit of SasA induction is likely multifold. First, damages to the cell wall is highly detrimental during growth since it can lead to cell lysis. Since (p)ppGpp accumulation and its associated depletion of GTP allows rapid and coordinated control of growth-determining processes such as ribosome synthesis (Liu et al., 2015a), connecting cell wall status to the stringent response likely increases survival in response to cell wall damages. Secondly, SasA expression is under the regulation of σ^M^/σ^W^ regulon along with a repertoire of other genes responsible for cell wall synthesis and division (Cao et al., 2002; Libby et al., 2019), thus allowing complementary response to cell wall stresses. Thirdly, many naturally existing antibiotics target the bacterial cell wall and can be produced by microbes occupying the same physiological niche. An example is the soil bacterium *B. subtilis* which co-exist with other cell wall antibiotic-producing *Bacillus* species. The presence of SasA-mediated response likely enables the bacteria to sense and survive emerging antibiotic assault to increase their competitive fitness.

## Data Availability Statement

The raw data supporting the conclusions of this article will be made available by the authors, without undue reservation.

## Author Contributions

DF and JW designed the study. DF and JY performed the experiments and data analysis. DS and DA-N provided the operation and technical assistance of the LC and MS instrument. DF, JY, and JW discussed the findings and wrote the manuscript. All authors contributed to the article and approved the submitted version.

## Conflict of Interest

The authors declare that the research was conducted in the absence of any commercial or financial relationships that could be construed as a potential conflict of interest.

## Supplementary Material

FIGURE S1 Induction of SasA expression does not result in loss of cell viability. Culture aliquots were taken over time after IPTG induction and plated on LB plates without IPTG for colony counts to monitor cell viability. Data shown are mean CFU/mL. Error bars represent SD. *n* = 2.

FIGURE S2 Metabolomic changes mediated by SasA. Heat map of metabolite changes in cells after *sasA* or *sasA*^*D87G*^ expression. Numbers indicate mean fold-change in binary logarithm relative to untreated cells. *n* = 2.

FIGURE S3 Hypoxanthine levels before and after *sasA* or *sasA*^*D87G*^ expression. Levels of hypoxanthine (HPX) before and after induction of *sasA* or *sasA*^*D87G*^ expression. UT: untreated, Induced: after induction. Data shown are LC/MS ion counts normalized to OD_600_. Error bars indicate SD. *n* = 2. ^∗∗^*p* < 0.01, ^∗^*p* < 0.05, ns: not significant (Student’s *t*-test).

FIGURE S4 Biosynthesis pathway of AppppA. AppppA is synthesized by a two-step reaction catalyzed by aminoacyl-tRNA syntheatse (AA-tRNA synthetase) using ATP as substrates. In the presence of ATP, amino acid (AA) is first adenylated by AA-tRNA synthetase to generate amino acid-AMP (AA-AMP). The AMP moiety in AA-AMP is then transferred to another ATP molecule to generate AppppA.
