# Supplementary material for: Small Alarmone Synthetase SasA Expression Leads to Concomitant Accumulation of pGpp, ppApp, and AppppA in Bacillus subtilis
Source: Front Microbiol. 2020 Sep 2;11:2083. doi: 10.3389/fmicb.2020.02083 (PMC7492591; doi:10.3389/fmicb.2020.02083)
Supplement: FIGURE S1 — Induction of SasA expression does not result in loss of cell viability. Culture aliquots were taken over time after IPTG induction and plated on LB plates without IPTG for colony counts to monitor cell viability. Data shown are mean CFU/mL. Error bars represent SD. n = 2. [file Data_Sheet_1.PDF]

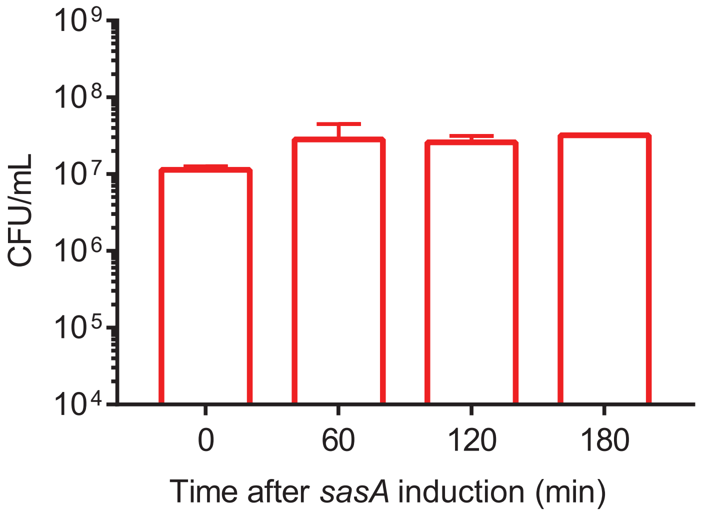

Supplementary figure 1. Induction of SasA expression does not result in loss of cell viability. Culture aliquots were taken over time after IPTG induction and plated on LB plates without IPTG for colony counts to monitor cell viability. Data shown are mean CFU/mL. Error bars represent SD. n = 2.

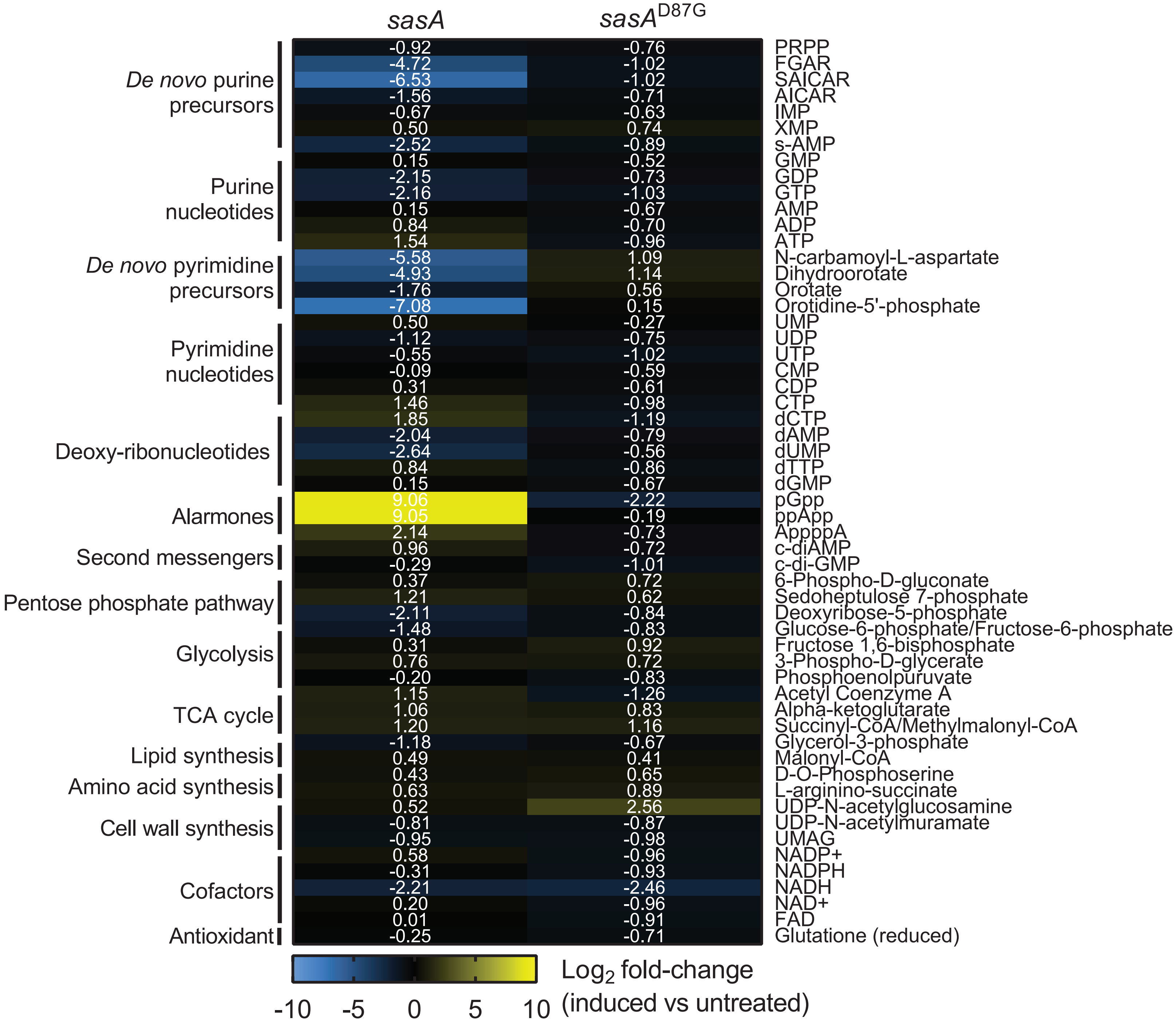

Supplementary figure 2. Metabolomic changes mediated by SasA. Heat map of metabolite changes in cells after *sasA* or *sasA*^{D87G} expression. Numbers indicate mean fold-change in binary logarithm relative to untreated cells. n = 2.

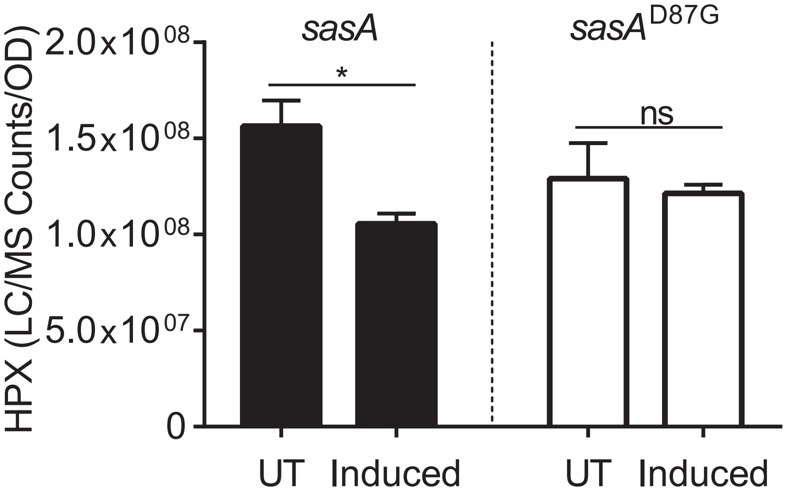

Supplementary figure 3. Hypoxanthine levels before and after *sasA* or *sasA*^{D87G} expression. Levels of hypoxanthine (HPX) before and after induction of *sasA* or *sasA*^{D87G} expression. UT: untreated, Induced: after induction. Data shown are LC/MS ion counts normalized to OD_{600}. Error bars indicate SD. n = 2. **: $p < 0.01$, *:$p < 0.05$, ns: not significant (Student's *t* test).

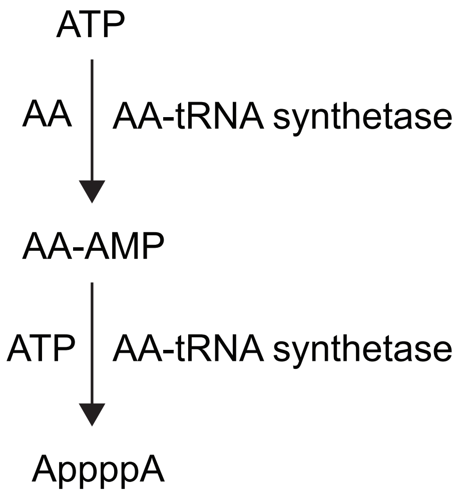

Supplementary figure 4. Biosynthesis pathway of AppppA. AppppA is synthesized by a two-step reaction catalyzed by aminoacyl-tRNA syntheatse (AA-tRNA synthetase) using ATP as substrates. In the presence of ATP, amino acid (AA) is first adenylated by AA-tRNA synthetase to generate amino acid-AMP (AA-AMP). The AMP moiety in AA-AMP is then transferred to another ATP molecule to generate AppppA.
